# Supplementary material for: CTCF loss has limited effects on global genome architecture in Drosophila despite critical regulatory functions
Source: Nat Commun. 2021 Feb 12;12:1011. doi: 10.1038/s41467-021-21366-2 (PMC7880997; doi:10.1038/s41467-021-21366-2)
Supplement: Supplementary file 1 — Supplementary Information [file 41467_2021_21366_MOESM1_ESM.pdf]

CTCF loss has limited effects on global genome architecture in *Drosophila* despite critical regulatory functions

SUPPLEMENTARY INFORMATION

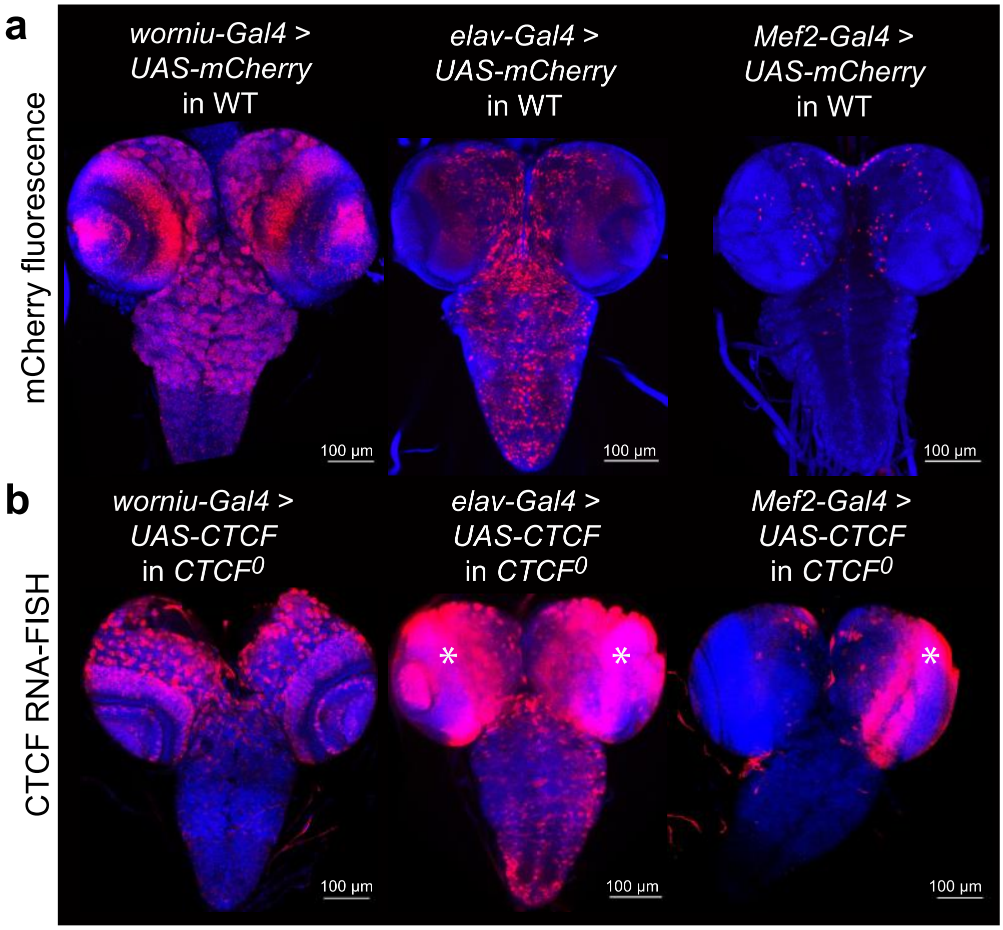

Supplementary Figure 1: Tissue-specific rescue of *CTCF*^{0} mutants.

(a) Wildtype third instar larval central nervous systems of animals expressing *UAS-mCherry* (red) under the control of Gal4 drivers used in Fig.1 that are active in neural stem cells (*worniu-Gal4*), mature neurons (*elav-Gal4*) or muscle (*Mef2-Gal4*). Gal4-expressing cells are marked by mCherry direct immunofluorescence. Scale bars 100 µm.

(b) Third instar larval central nervous systems of *CTCF*^{0} mutants analyzed in Fig. 1c-d, in which *UAS-CTCF* was expressed in restricted cells under the control of Gal4 drivers. Samples were labeled by RNA-FISH with an antisense probe to *CTCF* mRNA. White asterisks mark variable non-specific signal visible in the optic lobes of some *CTCF*^{0} mutants, also those without a *UAS-CTCF* transgene. Scale bars 100 µm.

Supplementary Figure 2

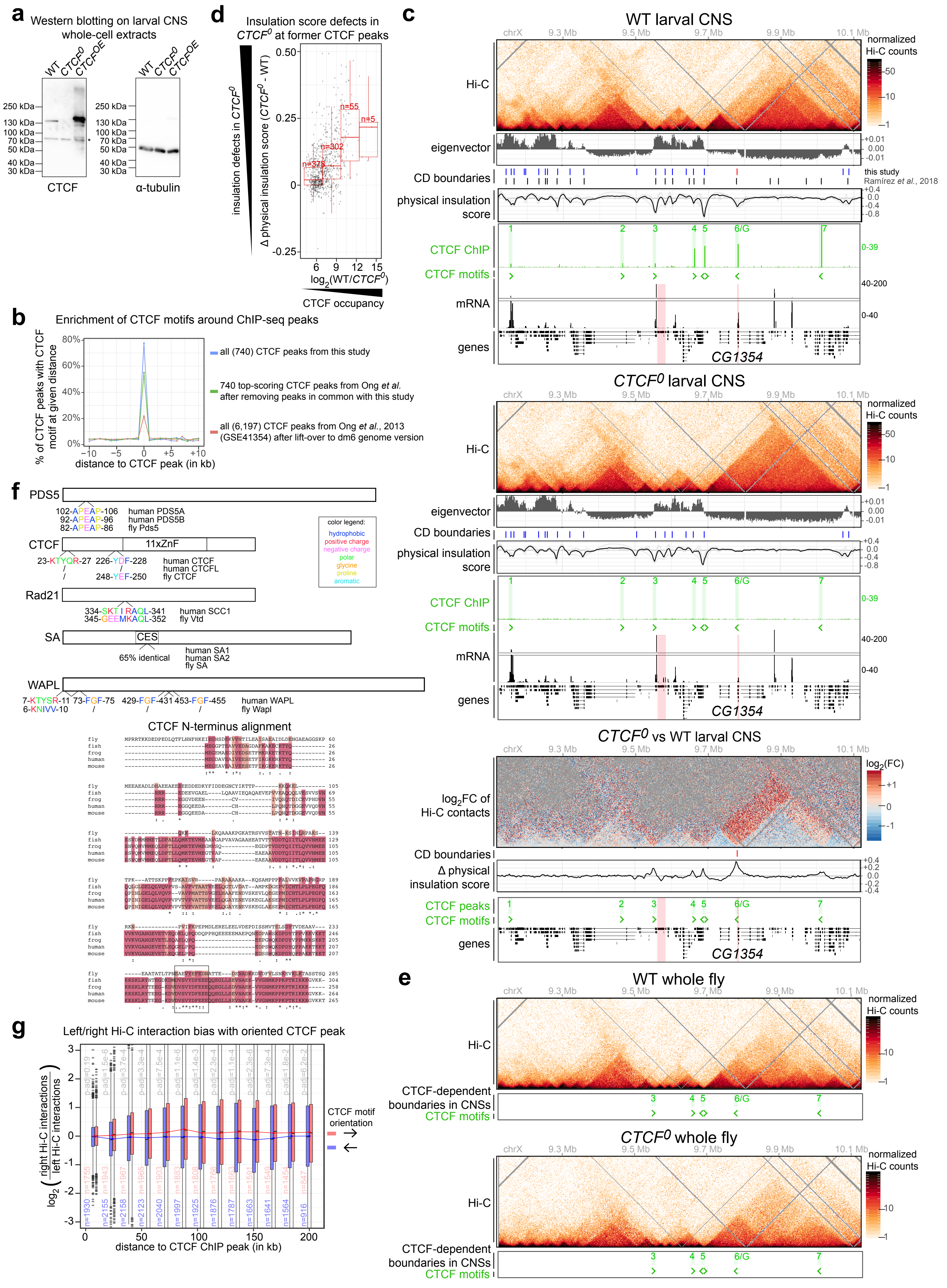

Supplementary Figure 2: Characterization of CTCF antibody and CTCF-dependent contact domain boundaries.

(a) Western blotting of whole-cell extracts from WT, *CTCF⁰* and CTCF-overexpressing (*CTCF^OE*) larval CNSs probed with anti-CTCF (asterisk marks cross-reacting band), then with anti-alpha-tubulin to verify equal loading of each extract.

(b) Percentage of CTCF peaks from this or a published study[1] with at least one CTCF motif (JASPAR motif MA0531.1) at a given distance.

(c) Example locus like Fig. 2c additionally displaying mRNA-seq tracks (genes differentially expressed in *CTCF⁰* highlighted). CTCF-dependent boundaries are observed near genes with unchanged (peak 7), decreased (*CG1354* near peak 6/G) or increased (peak 3) expression in *CTCF⁰* CNSs.

(d) Physical insulation score differences measured in *CTCF⁰* minus WT Hi-C maps as a function of CTCF occupancy measured by ChIP-seq [$\log_2$(WT/*CTCF⁰*)] for each CTCF peak (dots). Box plots of indicated n CTCF peaks binned by ChIP occupancy are overlaid. Box plots in d and g: center line, median; box limits, upper and lower quartiles; whiskers, 1.5x interquartile ranges; points, outliers.

(e) Hi-C maps like (c) generated from single whole-bodied WT or *CTCF⁰* flies. CTCF peaks 3-7 at which boundary defects were detected in *CTCF⁰* larval CNS Hi-C maps are marked.

(f) Fly and human CTCF and cohesin subunits and regulators implicated in TAD boundary formation. DNA-bound CTCF zinc-fingers (ZnF) form a semi-permeable barrier to loop-extruding cohesin in mammals[2]. Human CTCF YDF and fly CTCF YEF bind to the conserved essential surface in cohesin[3] (Fig. 2g). Mammalian CTCF KTYQR (similar to WAPL KTYSR) binds to PDS5A APEAP[2]; but fly CTCF lacks this motif and does not co-purify with Pds5 in vivo (Supplementary Fig. 5a). Human CTCF binds to cohesin competitively with WAPL (possibly via WAPL FGF) in vitro[3]. (Bottom) Clustal Omega alignment of fly and vertebrate (fish, frog, human, mouse) CTCF N-termini.

(g) Box plots of $\log_2$ ratios (n indicated in the figure) of right-over-left Hi-C interactions in WT established by forward (red) or reverse (blue) pointing CTCF motifs, at increasing distances from the CTCF peak (in 16 kb bins). Colored lines connect means. $\log_2$ ratios between forward and reverse pointing CTCF motifs were significantly different (indicated adjusted p-values <0.05) between 16 and 192 kb (two-sided Wilcoxon rank-sum test with Benjamini-Hochberg multiple testing correction).

Supplementary Figure 3

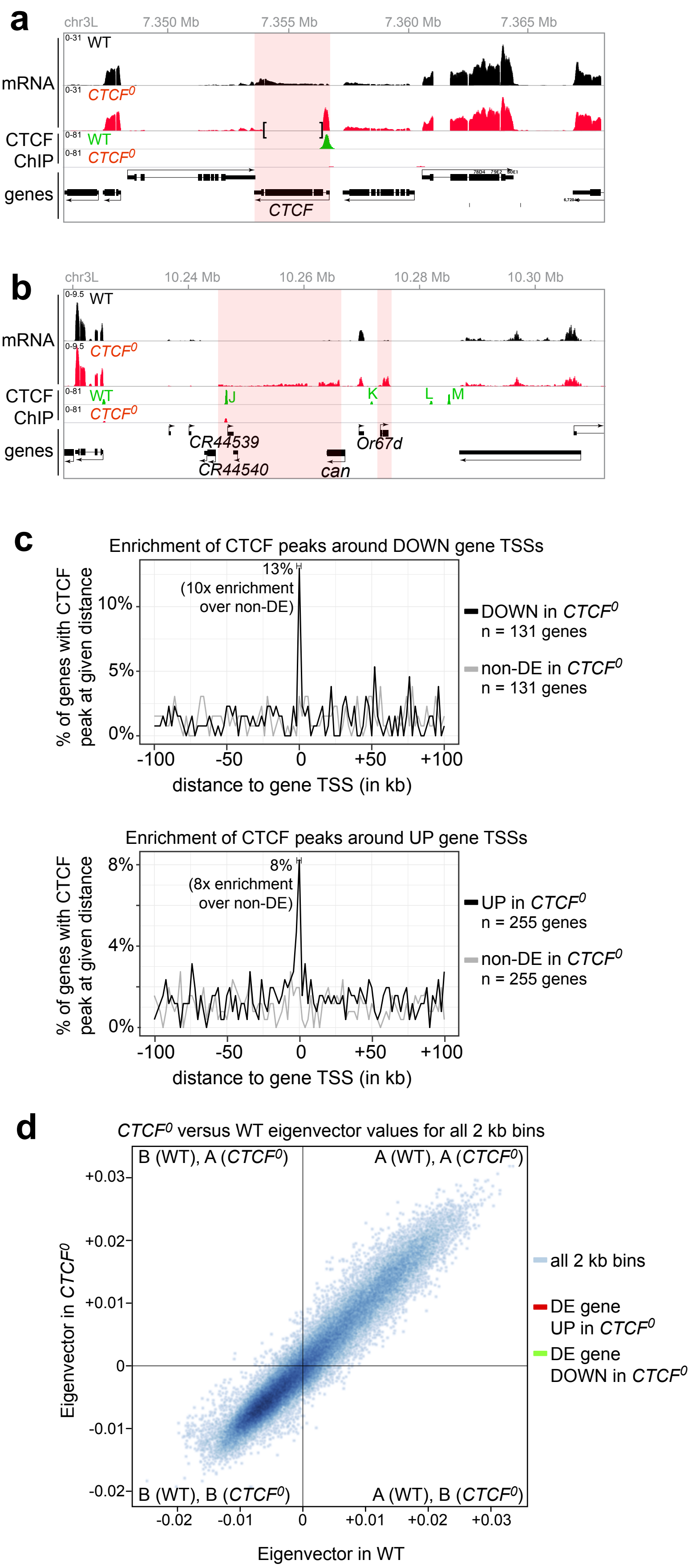

Supplementary Figure 3: Transcriptional defects in *CTCF⁰* mutant CNSs.

(a) Like Figs. 3b-d for the *CTCF* locus. The deleted region in $CTCF^0$ mutants is bracketed. Increased transcription of *CTCF* 5'UTR in $CTCF^0$ mutants could be due to potential CTCF-autoregulation or driven by 3xP3 regulatory sequences of the knocked-in *DsRed* selection marker [4,5].

(b) As above for a locus harboring a cryptic transcript (transcribed from left-to-right) in $CTCF^0$ CNSs, and *Or67d*.

(c) (Top) Percentage (in y) of n = 131 DE genes with decreased expression in $CTCF^0$ larval CNSs (black) or n = 131 randomly sampled expression-level-matched non-DE genes (grey) with at least one of 740 CTCF peaks at a given distance (per 2 kb bins) around the gene TSS, measured in the direction of transcription (in x). 13% of DOWN genes have at least one CTCF peak within ±1 kb of their TSS, which is 10-fold higher than the average enrichment at the sampled non-DE genes. (Bottom) Percentage (in y) of n = 255 DE genes with increased expression in $CTCF^0$ larval CNSs (black) or n = 255 randomly sampled expression-level-matched non-DE genes (grey) with at least one of 740 CTCF peaks at a given distance (per 2 kb bins) around the gene TSS, measured in the direction of transcription (in x). 8% of UP genes have at least one CTCF peak within ±1 kb of their TSS, which is 8-fold higher than the average enrichment at the sampled non-DE genes. 6 out of 392 DE genes were omitted from these analyses because they overlapped blacklisted regions in the CTCF ChIP-seq analysis [6].

(d) Eigenvector values in $CTCF^0$ mutants (in y) versus WT (in x) for every 2 kb bin of chromosomes 2, 3 and X (density plot in blue), with bins overlapping DE gene TSSs highlighted in red (for genes with increased expression in $CTCF^0$) or green (for genes with decreased expression in $CTCF^0$). Bins with positive eigenvector values are in the A (active) compartment, those with negative eigenvector values are in the B (inactive) compartment. Bins in the top left and bottom right quadrants are considered to be located in opposite compartments in $CTCF^0$ mutants relative to WT.

Supplementary Figure 4

a

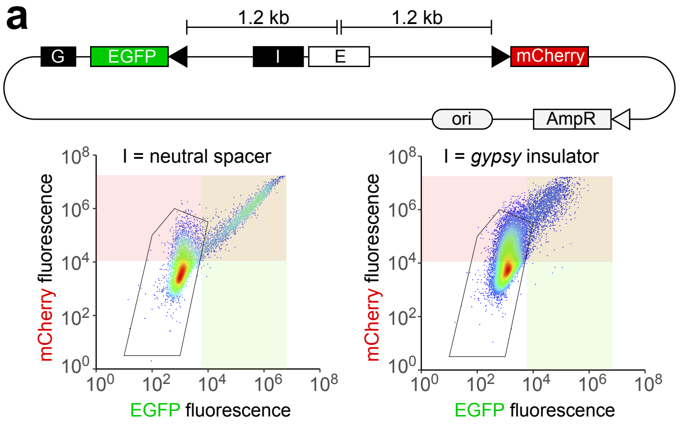

b

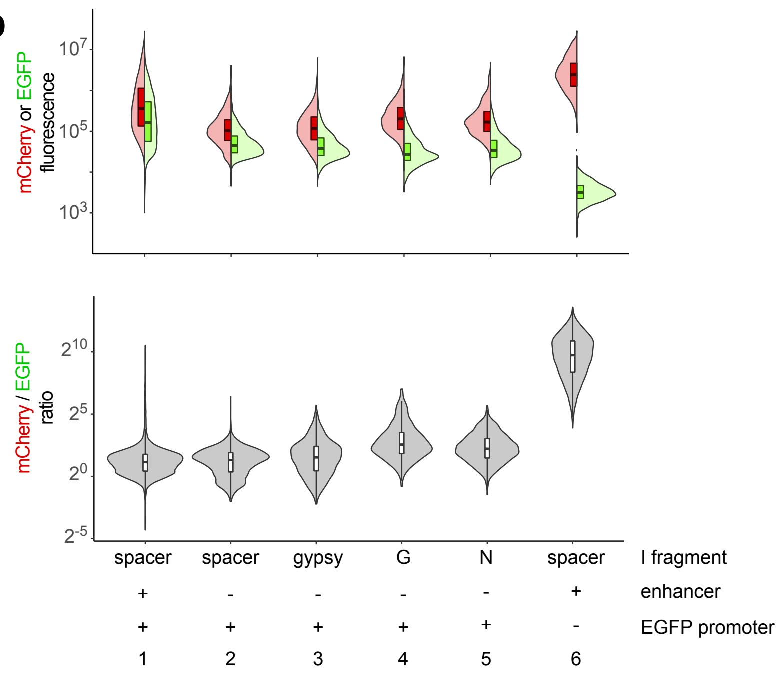

c

label	dm6 coordinates	size	close to differentially expressed gene in $CTCF^0$	position relative to closest gene	distance from fragment center to closest anotated TSS	# matches to CTCF motif	CTCF-dependent boundary in larval CNS?
A	chr2L:6684687-6684980	294 bp	*Tsp*	overlaps TTS	467 bp	1	partially CTCF-dependent
B	chr2L:6557352-6557697	346 bp	*IFT52*	overlaps TSS	22 bp	1	partially CTCF-dependent
C	chr2L:602317-602729	411 bp	*CG13689*	upstream of TSS	291 bp	3	strictly CTCF-dependent
D	chr2L:17784631-17785007	377 bp	none	downstream of TTS	2,151 bp	2	strictly CTCF-dependent
E	chr2R:6151039-6151512	474 bp	none	intron	5,853 bp	7	partially CTCF-dependent
F	chr2L:7534948-7535274	327 bp	none	intron	4,512 bp	3	strictly CTCF-dependent
G	chrX:9778493-9778804	312 bp	*CG1354*	upstream of TSS	96 bp	1	strictly CTCF-dependent
H	chr2R:19826269-19826627	359 bp	cryptic transcript	overlaps TSS	130 bp	0	strictly CTCF-dependent
I	chr2L:6797475-6797786	312 bp	none	intron	563 bp	2	strictly CTCF-dependent
J	chr3L:10246350-10246920	571 bp	*can*	upstream of TSS	1,843 bp	2	partially CTCF-dependent
K	chr3L:10271587-10271968	382 bp	*can*	upstream of TSS	1,412 bp	1	no boundary
L	chr3L:10281692-10282362	671 bp	*can*	downstream of TTS	7,318 bp	1	no boundary
M	chr3L:10284788-10285203	579 bp	*can*	downstream of TTS	10,000 bp	1	no boundary
N	chr3R:29251563-29251840	278 bp	*SP1029*	overlaps TSS	3,623 bp	2	strictly CTCF-dependent

d

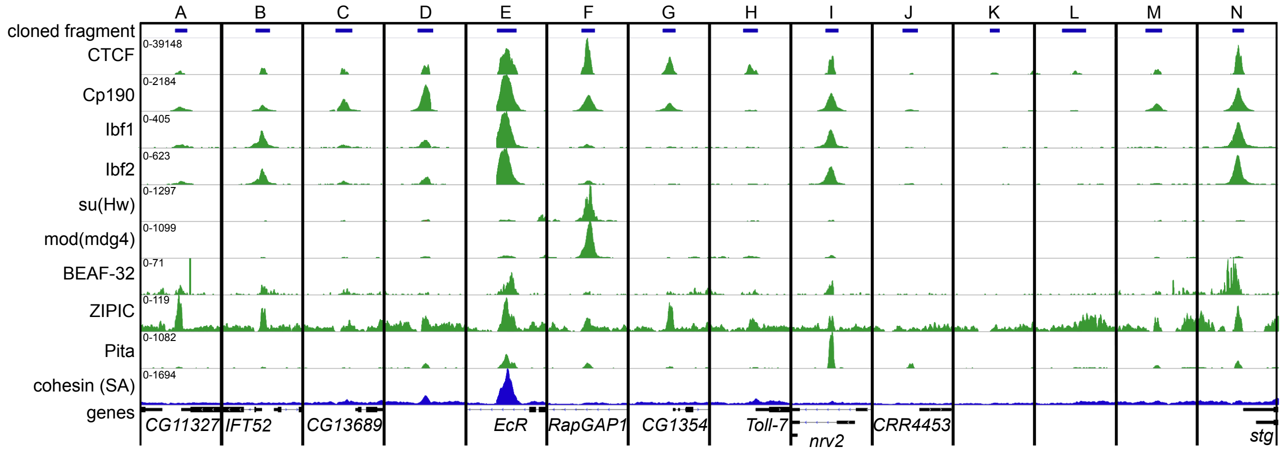

Supplementary Figure 4: Insulator reporter assay.

(a) Scatter plots of EGFP and mCherry fluorescence intensities in S2 cells transiently transfected with reporters with no insulator (left) or *gypsy* (right) cloned as I fragments. Gated untransfected cells (polygon) were excluded in Figs. 4b-c and in Supplementary Fig. 4b. Ranges of EGFP and mCherry fluorescence values considered to be above background (when outside of the black polygon) are shaded in green and red.

(b) Split violin plots (thick lines mark medians, boxes mark interquartile ranges) of mCherry (left) and EGFP (right) fluorescence intensities measured in thousands of single S2 cells (merged biological duplicates) transiently transfected with reporters with or without EGFP promoters or enhancers, and with the indicated I fragments. mCherry-to-EGFP ratios ($\log_2$ values) in single cells are shown blow. Reporters in lanes 1 (with promoter and enhancer), 2 (with promoter but no enhancer) and 6 (no promoter and no enhancer) reveal enhancer-activated, basal and background EGFP fluorescence levels. Reporters shown in lanes 2-5 lack an enhancer and differ by the cloned I fragment.

(c) For each cloned CTCF peak (labeled A-N in all figures) tested in Fig. 4c: dm6 coordinates; size; name of the nearby gene if this gene was differentially expressed in *CTCF⁰* mutant CNSs; position of the CTCF peak relative to the closest gene (irrespective of whether the gene is differentially expressed in *CTCF⁰* mutants or not); distance from the center of the cloned CTCF peak to the closest annotated TSS; number of matches to the Jaspar insect CTCF motif (MA0531.1); and whether this CTCF peak overlapped a CTCF-dependent boundary in larval CNSs. (TSS: transcription start site, TTS: transcription termination site)

(d) Published ChIP-seq profiles in S2 cells of indicated insulator-binding proteins and of cohesin SA on fragments A-N. The following datasets were re-mapped and visualized: CTCF, mod(mdg4) and su(Hw) from GSE41354[1]; Cp190, Ibf1 and Ibf2 from GSE47559[7]; Pita and ZIPIC from GSE54337[8]; BEAF-32 from GSE52962[9] and dSA from GSE85191[10]. Scales show total counts.

Supplementary Figure 5

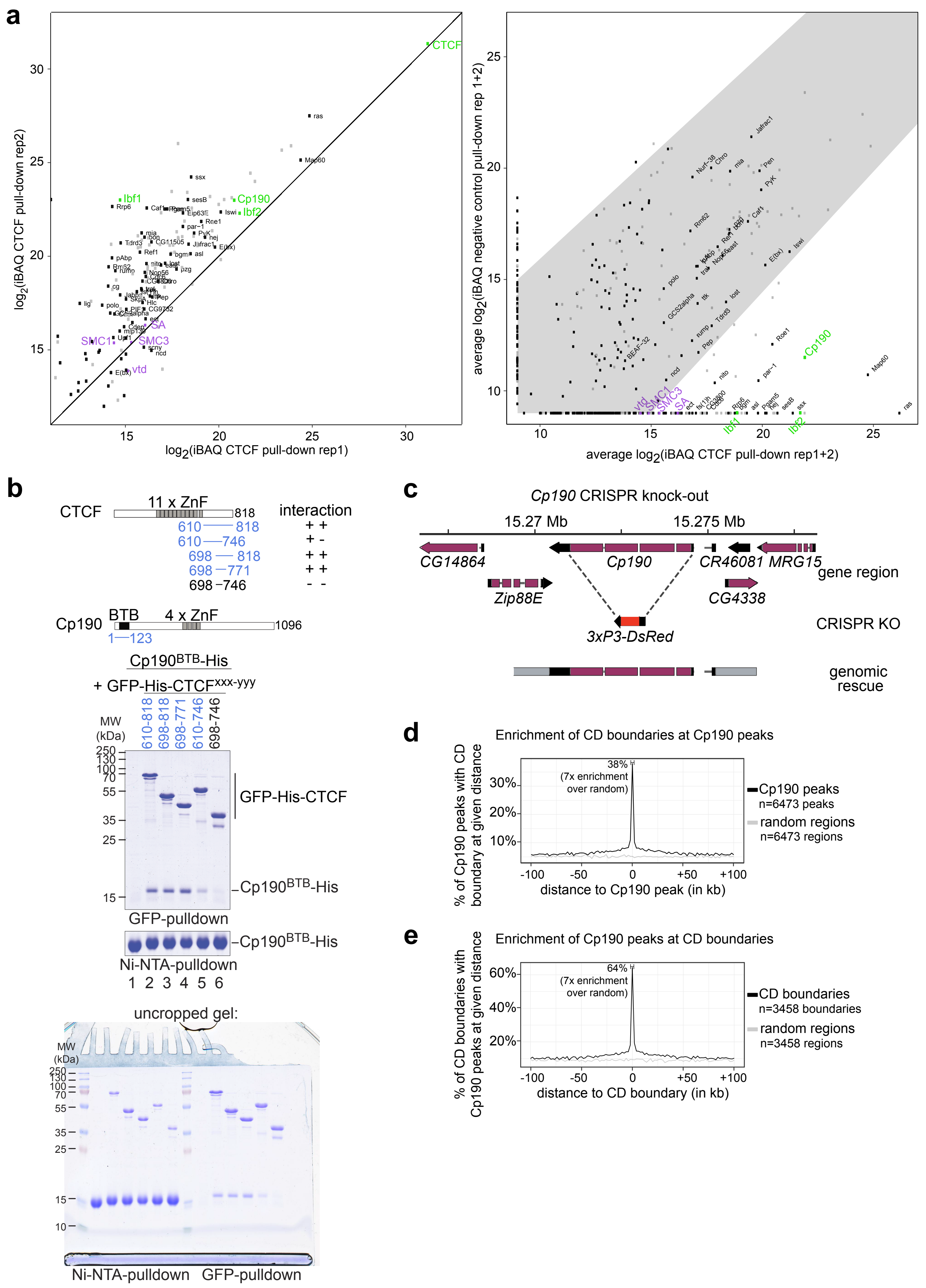

Supplementary Figure 5: CTCF stably binds to the pervasive boundary-associated factor Cp190.

(a) Proteins identified by mass spectrometry with indicated log_2 intensity-based absolute quantification (iBAQ) values. Known insulator-binding proteins (green) and cohesin subunits (purple) are indicated; abundant likely common contaminants are marked by grey dots (see Source Data). (Left) Proteins with iBAQ values >11 reproducibly co-purified with recombinant GFP-tagged CTCF N-terminus mixed with *Drosophila* embryonic nuclear extracts in biological duplicates. CTCF bait was added in excess to extracts and is disproportionately abundant in the pull-down. (Right) Average log_2 iBAQ values from biological duplicates of CTCF pull-downs or negative control pull-downs using recombinant GFP. Proteins not enriched by more than 64-fold (6 log_2 iBAQ units) in the CTCF pull-down relative to the negative control are shaded in grey.

(b) GFP-His-tagged CTCF constructs were co-expressed with His-tagged $Cp190^{BTB}$ in bacteria (blue fragments interacted, black did not). Extracts were split in half and subjected to GFP or Ni-NTA pull-downs (to control for similar amounts of $Cp190^{BTB}$ in each extract). $CTCF^{698\text{-}771}$ was the smallest fragment that retained $Cp190^{BTB}$ with similar efficiency as CTCF's entire C-terminal domain. Further C-terminally truncated $CTCF^{610\text{-}746}$ retained $Cp190^{BTB}$ more weakly, and further N-terminally truncated $CTCF^{698\text{-}746}$ bound even more weakly. The uncropped gel is below.

(c) *Cp190* extended gene region with coding (purple) and noncoding (black) exons and introns (lines). In *Cp190^KO^* mutants, a *DsRed* selection marker replaces *Cp190* open reading frame. $Cp190^{0}$ mutants were generated by excising (with FLP recombinase) the indicated FRT-flanked genomic rescue fragment from germlines of conditionally rescued $Cp190^{KO}$ mothers and fathers.

(d) Percentage of n=6473 Cp190 peaks in WT with at least one CD boundary in WT at a given distance (per 2 kb bins). Enrichment of CD boundaries around the same number of random positions (grey) is shown as control.

(e) Percentage of n=3458 CD boundaries in WT with at least one Cp190 peak in WT at a given distance (per 2 kb bins). Enrichment of Cp190 peaks around the same number of random positions (grey) is shown as control.

Supplementary Figure 6

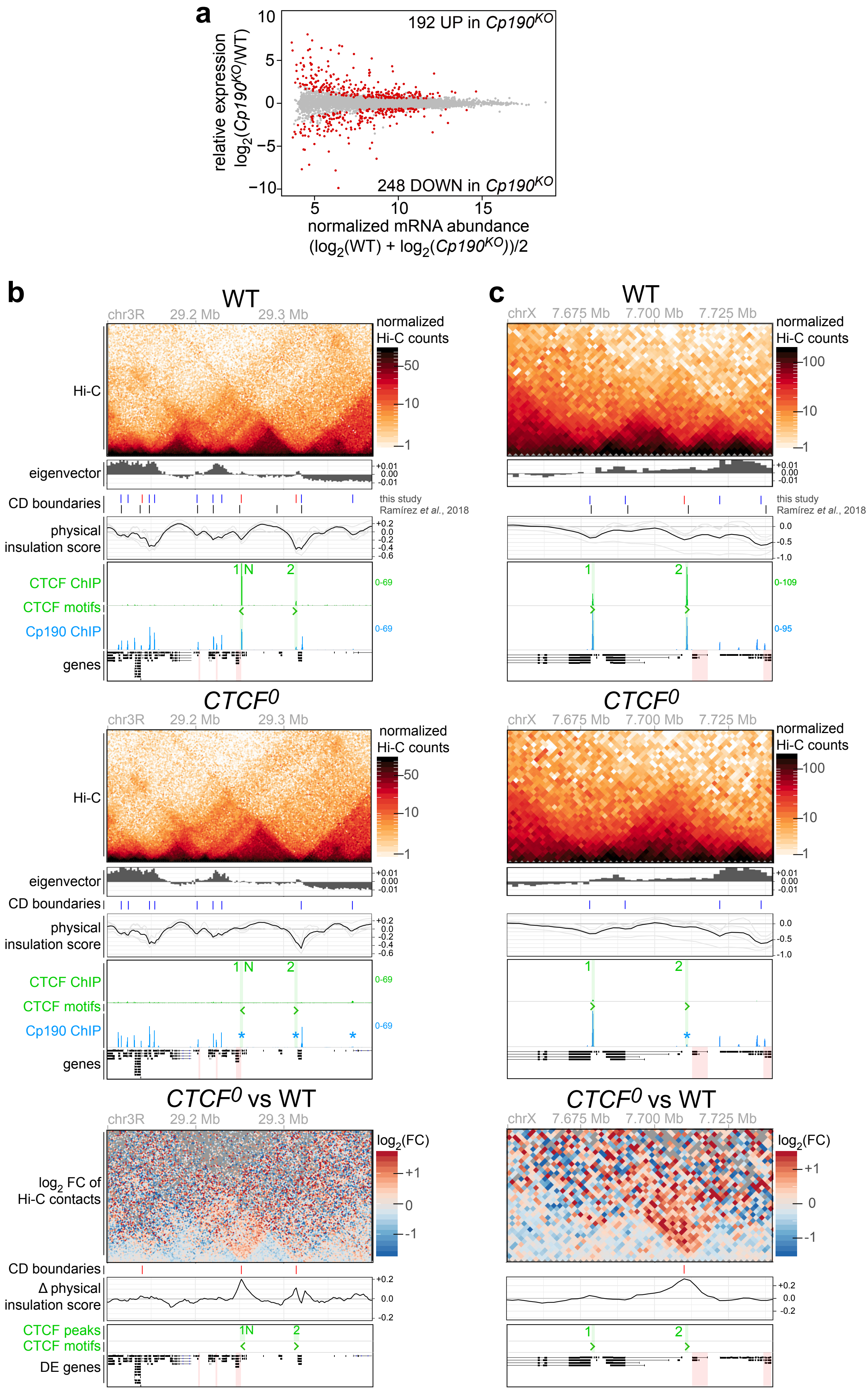

Supplementary Figure 6: Transcriptional misregulation in *Cp190^{KO}* CNSs and Hi-C maps of loci from Fig. 6.

(a) RNA-seq MA plot of *Cp190^{KO}* versus WT larval CNSs with mean abundance (in x) plotted as a function of enrichment (in y). DE genes (padj < 0.05 and |fold change|>1.5) are red.

(b) *SP1029* gene region shown in Fig. 6b with Hi-C maps, eigenvector values (positive for A compartment, negative for B compartment), CD boundaries from this study and a Hi-C study in cultured cells[11], physical insulation score (calculated with different window sizes in grey, average in black), CTCF ChIP-seq (CTCF peaks highlighted and numbered), CTCF motif orientations in DNA, Cp190 ChIP-seq (asterisks mark Cp190 peaks in *CTCF^0* mutants with reduced occupancy relative to WT revealed by differential analysis), and gene tracks (differentially expressed genes in *CTCF^0* relative to WT shaded in red) in WT (top) and *CTCF^0* (middle) larval CNSs. (Below) Differential (*CTCF^0* minus WT) Hi-C maps and physical insulation score.

(c) Same as (b) but for the *CG15478* extended gene region shown in Fig. 6d.

Supplementary Table 1: Quality metrics of Hi-C reads.

Hi-C sample	total reads	interchr	intrachr	intrachr <20 kb	intrachr >20 kb
WT larval brain (combined replicates)	200,000,000 (downsampled)	24,618,605	175,381,395	50,788,347	124,593,048
$CTCF^{0}$ larval brain (combined replicates)	200,000,000 (downsampled)	27,076,150	172,923,850	47,787,104	125,136,746
WT whole fly (single replicate)	11,0261,440	22201387	88060053	26894987	124,593,048
$CTCF^{0}$ whole fly (single replicate)	83,302,985	19,068,061	64,234,924	16,155,686	48,079,238

Supplementary Table 2: Contact domain (CD) boundary counts.

(Column 1) Total CD boundaries called in both WT and *CTCF*^{0} mutants (common), or only in WT, or only in *CTCF*^{0} mutant larval CNS Hi-C maps. CD boundaries were split into those with (columns 2 and 3) or without (columns 4 and 5) a CTCF peak within ±2 kb (the resolution at which CD boundaries were called). Strongly affected CD boundaries (columns 3 and 5) were defined as having a physical insulation score difference between *CTCF*^{0} minus WT Hi-C maps > 0.1 (weaker boundary in *CTCF*^{0} relative to WT) for boundaries only called in WT, or < -0.1 (stronger boundary in *CTCF*^{0} relative to WT) for boundaries only called in *CTCF*^{0}, or an absolute value > 0.1 for common boundaries.

	1	2	3	4	5
	All CD boundaries	CD boundaries with CTCF peak ±2kb		CD boundaries without CTCF peak ±2 kb	
		all	strongly affected	all	strongly affected
Common	2891	218	75	2673	51
Only in WT	567	125	89	442	46
Only in *CTCF*^{0}	512	6	1	506	22
Total	3970	349	165	3621	119

Supplementary Table 3: Interactive links to browse Hi-C and ChIP-seq data on Juicebox.

Differential Hi-C maps of WT (A) and *CTCF*^{0} (B) larval CNSs are displayed for the indicated genomic intervals (1 Mb each). Additional tracks are: CTCF ChIP-seq peaks in WT (track 1), CTCF peaks intersected with underlying motifs in forward (green) or reverse (red) orientations (track 2), Cp190 ChIP-seq peaks in WT (track 3), and Cp190 ChIP-seq signal that is differentially enriched in *CTCF*^{0} mutants relative to WT (track 4).

dm6 genomic coordinates	url
NT_033779.5:1-1,000,000	http://bit.ly/2TM42hd
NT_033779.5:1,000,000-2,000,000	http://bit.ly/2VSG0DV
NT_033779.5:5,000,000-6,000,000	http://bit.ly/2xgSjjn
NT_033779.5:6,000,000-7,000,000	http://bit.ly/2PTGFRO
NT_033779.5:7,000,000-8,000,000	http://bit.ly/2PQQ3FK
NT_033779.5:14,000,000-15,000,000	http://bit.ly/3avWeHl
NT_033778.4:8,000,000-9,000,000	http://bit.ly/2TnejBz
NT_037436.4:3,000,000-4,000,000	http://bit.ly/2PU06tU
NT_037436.4:6,000,000-7,000,000	http://bit.ly/3awLuZ7
NT_037436.4:14,000,000-15,000,000	http://bit.ly/3aEI5I5
NT_033777.3:4,800,000-5,800,000	http://bit.ly/2VQiLud
NT_033777.3:7,000,000-8,000,000	http://bit.ly/3aydD1Z
NT_033777.3:14,000,000-15,000,000	http://bit.ly/3cAfmpr
NT_033777.3:16,000,000-17,000,000	http://bit.ly/2PUXaNH
NT_033777.3:28,500,000-29,500,000	http://bit.ly/2VMq1aJ
NC_004354.4:9,500,000-10,500,000	http://bit.ly/2vAMyN6
NC_004354.4:15,000,000-16,000,000	http://bit.ly/2TIN2sl
NC_004354.4:17,000,000-18,000,000	http://bit.ly/2vItGvm
NC_004354.4:20,500,000-21,500,000	http://bit.ly/2VSxBke

Supplementary References

1. Ong, C.-T., Van Bortle, K., Ramos, E. & Corces, V. G. Poly(ADP-ribosyl)ation Regulates Insulator Function and Intrachromosomal Interactions in Drosophila. *Cell* **155**, 148–159 (2013).

2. Nora, E. P. *et al.* Molecular basis of CTCF binding polarity in genome folding. *Nat Commun* **11**, 5612 (2020).

3. Li, Y. *et al.* The structural basis for cohesin–CTCF-anchored loops. *Nature* **578**, 1–9 (2020).

4. Gratz, S. J., Harrison, M. M., Wildonger, J. & O'Connor-Giles, K. M. Precise Genome Editing of Drosophila with CRISPR RNA-Guided Cas9. *Methods in molecular biology (Clifton, N.J.)* **1311**, 335–348 (2015).

5. Gambetta, M. C. & Furlong, E. E. M. The Insulator Protein CTCF Is Required for Correct Hox Gene Expression, but Not for Embryonic Development in Drosophila. *Genetics* **210**, 129–136 (2018).

6. Amemiya, H. M., Kundaje, A. & Boyle, A. P. The ENCODE Blacklist: Identification of Problematic Regions of the Genome. *Sci Rep-uk* **9**, 9354 (2019).

7. Cuartero, S., Fresán, U., Reina, O., Planet, E. & Espinàs, M. L. Ibf1 and Ibf2 are novel CP190-interacting proteins required for insulator function. *The EMBO Journal* **33**, 637–647 (2014).

8. Maksimenko, O. *et al.* Two new insulator proteins, Pita and ZIPIC, target CP190 to chromatin. *Genome Research* **25**, 89–99 (2015).

9. Liang, J. *et al.* Chromatin Immunoprecipitation Indirect Peaks Highlight Long-Range Interactions of Insulator Proteins and Pol II Pausing. *Molecular Cell* **53**, 672–681 (2014).

10. Henriques, T. *et al.* Widespread transcriptional pausing and elongation control at enhancers. *Genes & development* **32**, 26–41 (2018).

11. Ramírez, F. *et al.* High-resolution TADs reveal DNA sequences underlying genome organization in flies. *Nature Communications* **9**, 189 (2018).
