# Supplementary material for: CTCF loss has limited effects on global genome architecture in Drosophila despite critical regulatory functions
Source: Nat Commun. 2021 Feb 12;12:1011. doi: 10.1038/s41467-021-21366-2 (PMC7880997; doi:10.1038/s41467-021-21366-2)
Supplement: Supplementary file 3 — Description of Additional Supplementary Files [file 41467_2021_21366_MOESM3_ESM.pdf]

Description of Additional Supplementary Files

Supplementary Data 1: Differential CTCF binding analysis (wildtype versus $CTCF^{0}$) with csaw, using Benjamini & Hochberg method to control the false discovery rate. Regions with false discovery rate (FDR)<0.01 and |log2(fold change)|>1 were considered as differential binding regions. CTCF peaks are sites with direction=up.

Supplementary Data 2: Physical insulation scores in wildtype and $CTCF^{0}$.

Supplementary Data 3: Contact domain boundaries found in wildtype and/or in $CTCF^{0}$ and associated physical insulation scores.

Supplementary Data 4: Differential RNA-seq analysis ($CTCF^{0}$ versus wildtype). Statistical significance of differential gene expression was measured using a Wald Test from DESeq2 package. P-values are two-tailed and adjusted for multiple testing using the Benjamini-Hochberg method.

Supplementary Data 5: First eigenvector and A/B compartments.

Supplementary Data 6: Differential Cp190 binding analysis (wildtype versus $Cp190^{KO}$) with csaw, using Benjamini & Hochberg method to control the false discovery rate. Regions with false discovery rate (FDR)<0.01 and |log2(fold change)|>1 were considered as differential binding regions. Cp190 peaks are sites with direction=up.

Supplementary Data 7: Differential Cp190 binding analysis (wildtype versus $CTCF^{0}$) with csaw, using Benjamini & Hochberg method to control the false discovery rate. Regions with false discovery rate (FDR)<0.01 and |log2(fold change)|>1 were considered as differential binding regions.

Supplementary Data 8: Differential Cp190 binding analysis ($CTCF^{0}$ versus $Cp190^{KO}$) with csaw, using Benjamini & Hochberg method to control the false discovery rate. Regions with false discovery rate (FDR)<0.01 and |log2(fold change)|>1 were considered as differential binding regions. Cp190 peaks in $CTCF^{0}$ are sites with direction=up.

Supplementary Data 9: Differential RNA-seq analysis ($Cp190^{KO}$ versus wildtype). Statistical significance of differential gene expression was measured using a Wald Test from DESeq2 package. P-values are two-tailed and adjusted for multiple testing using the Benjamini-Hochberg method.

Supplementary Data 10: List of primers.

Supplementary Movie 1: $CTCF^{KO}$ animals that hatched from the pupal case.

Supplementary Movie 2: $CTCF^{KO}$ animals rescued with a *CTCF* rescue transgene that was conditionally excised by Flp recombinase expressed under the control of a Gal4 driver active in neural stem cells (*worniu-Gal4*).

Supplementary Movie 3: $CTCF^{0}$ animals rescued with a *UAS-CTCF* rescue transgene conditionally expressed under the control of a Gal4 driver active in neural stem cells (*worniu-Gal4*).
