# Supplementary material for: CTCF loss has limited effects on global genome architecture in Drosophila despite critical regulatory functions
Source: Nat Commun. 2021 Feb 12;12:1011. doi: 10.1038/s41467-021-21366-2 (PMC7880997; doi:10.1038/s41467-021-21366-2)
Supplement: Supplementary file 17 — Reporting summary [file 41467_2021_21366_MOESM17_ESM.pdf]

nature research

Corresponding author(s): Erez Lieberman Aiden, Maria Cristina Gambetta

Last updated by author(s): Dec 12, 2020

Reporting Summary

Nature Research wishes to improve the reproducibility of the work that we publish. This form provides structure for consistency and transparency in reporting. For further information on Nature Research policies, see our Editorial Policies and the Editorial Policy Checklist.

Statistics

For all statistical analyses, confirm that the following items are present in the figure legend, table legend, main text, or Methods section.

n/a	Confirmed	
☐	☒	The exact sample size (n) for each experimental group/condition, given as a discrete number and unit of measurement
☐	☒	A statement on whether measurements were taken from distinct samples or whether the same sample was measured repeatedly
☐	☒	The statistical test(s) used AND whether they are one- or two-sided<br>*Only common tests should be described solely by name; describe more complex techniques in the Methods section.*
☒	☐	A description of all covariates tested
☐	☒	A description of any assumptions or corrections, such as tests of normality and adjustment for multiple comparisons
☐	☒	A full description of the statistical parameters including central tendency (e.g. means) or other basic estimates (e.g. regression coefficient) AND variation (e.g. standard deviation) or associated estimates of uncertainty (e.g. confidence intervals)
☐	☒	For null hypothesis testing, the test statistic (e.g. F, t, r) with confidence intervals, effect sizes, degrees of freedom and P value noted<br>*Give P values as exact values whenever suitable.*
☒	☐	For Bayesian analysis, information on the choice of priors and Markov chain Monte Carlo settings
☒	☐	For hierarchical and complex designs, identification of the appropriate level for tests and full reporting of outcomes
☐	☒	Estimates of effect sizes (e.g. Cohen's d, Pearson's r), indicating how they were calculated

Our web collection on statistics for biologists contains articles on many of the points above.

Software and code

Policy information about availability of computer code

Data collection	Mass spectrometry:<br>Xcalibur software v4.2 (Thermo Fisher)<br><br>ChIP-seq, Hi-C, RNA-seq:<br>Micmap v2.20200223 (Iseli et al, 2007): https://github.com/sib-swiss/micmap
Data analysis	All analyses were performed using published open source software and links that are also listed in the "Code availability" section.<br>Custom scripts are provided in https://github.com/gambettalab/kaushal2020/.<br><br>Mass spectrometry:<br>MaxQuant 1.6.3.4 (Cox et al. 2008, 2011)<br>Perseus v1.6.2.3 (Tyanova 2016)<br><br>Imaging (Western blotting and RNA-FISH):<br>Fiji v2.1.0/1.53f: https://imagej.net/Fiji/<br><br>Hi-C:<br>iced v0.5.2 (Servant et al, 2015): https://github.com/hiclib/iced<br>TopDom v0.0.2: https://github.com/jasminezhoulab/TopDom<br>Juicebox v1.5.1 (Durand et al, 2016): aidenlab.org/juicebox<br><br>ChIP-seq:

csaw v1.16.1 (Lun and Smyth, 2014, 2015): https://bioconductor.org/packages/release/bioc/html/csaw.html
edgeR v3.22.5: https://bioconductor.org/packages/release/bioc/html/edgeR.html
Eulerr v6.0.0 (Larsson 2019): https://cran.r-project.org/package=eulerr
bedtools multicov v2.29.2 (Quinlan 2010): https://bedtools.readthedocs.io/en/latest/

RNA-seq:
DEseq2 v1.22.2 (Love, Huber and Anders, 2014): https://bioconductor.org/packages/release/bioc/html/DESeq2.html
HTSeq v0.9.1 (Anders, Pyl and Huber, 2015): https://github.com/simon-anders/htseq
edgeR v3.22.5 (Robinson, McCarthy and Smyth, 2009): https://bioconductor.org/packages/release/bioc/html/edgeR.html

General:
R v3.5.1 (R Core Team 2018): https://www.R-project.org/
ggplot2 v3.1.0 (Wickham 2016): https://ggplot2.tidyverse.org/

Custom scripts to regenerate figures from the raw data provided in Source data are provided in: https://github.com/gambettalab/kaushal2020/

For manuscripts utilizing custom algorithms or software that are central to the research but not yet described in published literature, software must be made available to editors and reviewers. We strongly encourage code deposition in a community repository (e.g. GitHub). See the Nature Research guidelines for submitting code & software for further information.

Data

Policy information about availability of data

All manuscripts must include a data availability statement. This statement should provide the following information, where applicable:

- Accession codes, unique identifiers, or web links for publicly available datasets
- A list of figures that have associated raw data
- A description of any restrictions on data availability

All sequencing data (Hi-C, ChIP-seq, RNA-seq) that support the findings of this study were deposited in Gene Expression Omnibus with accession code GSE146752 (https://www.ncbi.nlm.nih.gov/geo/query/acc.cgi?acc=GSE146752). Hi-C maps are browsable on Juicebox (Supplementary Table 3). Mass spectrometry proteomics data were deposited to the ProteomeXchange Consortium via the PRIDE partner repository with the dataset identifier PXD019487 (https://www.ebi.ac.uk/pride/archive?keyword=PXD019487%20). Raw data for Figs. 1-6 is provided in Source Data and additional information is provided in Supplementary Data files 1-10.

Field-specific reporting

Please select the one below that is the best fit for your research. If you are not sure, read the appropriate sections before making your selection.

[x] Life sciences   [ ] Behavioural & social sciences   [ ] Ecological, evolutionary & environmental sciences

For a reference copy of the document with all sections, see nature.com/documents/nr-reporting-summary-flat.pdf

Life sciences study design

All studies must disclose on these points even when the disclosure is negative.

Sample size	For Drosophila viability tests performed in Figure 1, 100 animals were analyzed per genotype because clear differences between genotypes were visible already at this scale.<br>For RNA-FISH experiments shown in Supplementary Fig. 1b and Fig. 3e, 10 larvae were examined per genotype over two independent experiments and only representative phenotypes observed in all animals are shown. For RNA-FISH experiments shown in Figs. 6c and 6e, 50 embryos were examined per genotype over two independent RNA-FISH experiments and only representative phenotypes observed in all embryos of the same stage (11 hours post fertilization) are shown. These numbers were chosen because they revealed that phenotypes were reproducibly detected in all animals and because sample collection beyond this scale was rate-limiting as the mutant animals were obtained by genetic crosses (and hand-dissection for larval brains).<br>For ChIP-seq, Hi-C and RNA-seq experiments, 60 third instar larval brains were dissected on ice per replicate because this number allowed sufficient material to be amplified for next-generation sequencing library preparation with a limited number of PCR cycles to avoid over-amplification. This number was sufficient because all biological replicates were well correlated. Dissecting more samples could have compromised the quality of the samples because it would have required keeping the dissected organs on ice for many hours.
Data exclusions	No data was excluded from the analyses.
Replication	Next-generation sequencing datasets were generated and analyzed in biological triplicates (RNA-seq, Hi-C, CTCF ChIP-seq in wildtype) or duplicates (all remaining ChIP-seq datasets).<br>Protein purifications analyzed by mass spectrometry were performed and analyzed in biological duplicates.<br>All attempts at replication were successful except for one CTCF ChIP-seq in wildtype replicate that failed in the library preparation step. CTCF ChIP-seq in wildtype was then repeated to generate 2 new biological replicates that were successful, and analyses were performed with the combined successful triplicates (replicates 1, 2 and 3).
Randomization	Samples were grouped according to genotype (wildtype or various mutants).

Blinding	The investigators were not blinded during data collection as the biological groups (genotypes) were well defined and handled in parallel. Computational analysis was performed by data scientists different from the researchers who collected the data.

Reporting for specific materials, systems and methods

We require information from authors about some types of materials, experimental systems and methods used in many studies. Here, indicate whether each material, system or method listed is relevant to your study. If you are not sure if a list item applies to your research, read the appropriate section before selecting a response.

Materials & experimental systems

n/a	Involved in the study
☐	☒ Antibodies
☐	☒ Eukaryotic cell lines
☒	☐ Palaeontology and archaeology
☐	☒ Animals and other organisms
☒	☐ Human research participants
☒	☐ Clinical data
☒	☐ Dual use research of concern

Methods

n/a	Involved in the study
☐	☒ ChIP-seq
☐	☒ Flow cytometry
☒	☐ MRI-based neuroimaging

Antibodies

Antibodies used	Rabbit polyclonal antibodies against Drosophila CTCF and Cp190 proteins were generated for this study as described in the Methods. Mouse monoclonal anti-alpha-tubulin clone B-5-1-2 (Sigma T5168, lot 038M4813V) was used as loading control for Western blotting. Anti-digoxigenin-peroxidase (Roche 11207733910, lot 28557000) was used for RNA-FISH.
Validation	CTCF and Cp190 antibodies were validated by performing the following control experiments on CTCF and Cp190 mutant animals:<br>1/ CTCF WT ChIP-seq signals were globally lost in CTCF[0] mutants (Figs. 2,3,5,6 and Supplementary Figures 2,3,6);<br>2/ CTCF is specifically detected by Western blotting (Supplementary Figure 2a);<br>3/ Cp190 ChIP peaks detected in WT were lost or reduced in Cp190[KO] mutants (Fig. 6). Reduced Cp190 ChIP-seq signal in Cp190 [KO] mutants detected at some peaks could be expected maternally deposited Cp190 present in Cp190[KO] mutants. Cp190 ChIP peaks overlapping CTCF peaks detected in WT were specifically lost or reduced in CTCF[0] mutants (Figs. 5,6 and Supplementary Figure 6).

Eukaryotic cell lines

Policy information about cell lines

Cell line source(s)	Drosophila SL2 (Schneider's Drosophila Line 2) cells were originally purchased from ATCC (reference number CRL-1963) in 2006.
Authentication	The SL2 cell line was not authenticated because it is commercially available.
Mycoplasma contamination	The SL2 cell line tested negative for mycoplasma contamination by PCR upon receipt from the ATCC.
Commonly misidentified lines (See ICLAC register)	No cell lines from the ICLAC register were used.

Animals and other organisms

Policy information about studies involving animals; ARRIVE guidelines recommended for reporting animal research

Laboratory animals	Drosophila melanogaster w[1118] strain or mutant derivatives generated by CRISPR-Cas9 genome-editing were analyzed at embryo, third instar larva and adult stages as described in the Methods. Animals were not separated by sex, except for whole-fly Hi-C maps which were generated from females as described in the Methods.
Wild animals	No wild animals were used in this study.
Field-collected samples	No field-collected samples were used in this study.
Ethics oversight	This study did not require an ethical approval.

Note that full information on the approval of the study protocol must also be provided in the manuscript.

ChIP-seq

Data deposition

- [x] Confirm that both raw and final processed data have been deposited in a public database such as GEO.
- [x] Confirm that you have deposited or provided access to graph files (e.g. BED files) for the called peaks.

Data access links<br>*May remain private before publication.*	https://www.ncbi.nlm.nih.gov/geo/query/acc.cgi?acc=GSE146752
Files in database submission	GSM4405388 ChIP-seq_CTCF, wildtype, rep 1<br>GSM4966396 ChIP-seq_CTCF, wildtype, rep 2<br>GSM4966397 ChIP-seq_CTCF, wildtype, rep 3<br>GSM4405389 ChIP-seq_CTCF, CTCF[0] mutant, rep 1<br>GSM4405390 ChIP-seq_CTCF, CTCF[0] mutant, rep 2<br>GSM4405391 ChIP-seq_CTCF, Cp190[KO] mutant, rep1<br>GSM4405392 ChIP-seq_CTCF, Cp190[KO] mutant, rep2<br>GSM4405393 ChIP-seq_Cp190, wildtype, rep 1<br>GSM4405394 ChIP-seq_Cp190, wildtype, rep 2<br>GSM4405395 ChIP-seq_Cp190, CTCF[0] mutant, rep 1<br>GSM4405396 ChIP-seq_Cp190, CTCF[0] mutant, rep 2<br>GSM4405397 ChIP-seq_Cp190, Cp190[KO] mutant, rep 1<br>GSM4405398 ChIP-seq_Cp190, Cp190[KO] mutant, rep 2
Genome browser session<br>(e.g. UCSC)	The Hi-C and ChIP-seq data is browsable in Juicebox (as described in Supplementary Table S1):<br>http://bit.ly/2TM42hd<br>http://bit.ly/2VSG0DV<br>http://bit.ly/2xgSjjn<br>http://bit.ly/2PTGFRO<br>http://bit.ly/2PQQ3FK<br>http://bit.ly/3avWeHl<br>http://bit.ly/2TnejBz<br>http://bit.ly/2PU06tU<br>http://bit.ly/3awLuZ7<br>http://bit.ly/3aEI5I5<br>http://bit.ly/2VQiLud<br>http://bit.ly/3aydD1Z<br>http://bit.ly/3cAfmpr<br>http://bit.ly/2PUXaNH<br>http://bit.ly/2VMq1aJ<br>http://bit.ly/2vAMyN6<br>http://bit.ly/2TIN2sl<br>http://bit.ly/2vltGvm<br>http://bit.ly/2VSxBke

Methodology

Replicates	Each ChIP was performed in biological duplicates, except for CTCF ChIP-seq in wildtype that was performed in biological triplicates, and showed good agreement.
Sequencing depth	GSM4405388 ChIP-seq_CTCF, wildtype, rep 1 - 20972834 unique reads - 144bp, PE<br>GSM4966396 ChIP-seq_CTCF, wildtype, rep 2 - 26182228 unique reads - 144 bp, PE<br>GSM4966397 ChIP-seq_CTCF, wildtype, rep 3 - 27923954 unique reads - 144 bp, PE<br>GSM4405389 ChIP-seq_CTCF, CTCF[0] mutant, rep 1 - 19321710 unique reads - 144 bp, PE<br>GSM4405390 ChIP-seq_CTCF, CTCF[0] mutant, rep 2 - 22561508 unique reads - 144 bp, PE<br>GSM4405391 ChIP-seq_CTCF, Cp190[KO] mutant, rep1 - 20205568 unique reads - 144 bp, PE<br>GSM4405392 ChIP-seq_CTCF, Cp190[KO] mutant, rep2 - 21701536 unique reads - 144 bp, PE<br>GSM4405393 ChIP-seq_Cp190, wildtype, rep 1 - 23583410 unique reads - 144 bp, PE<br>GSM4405394 ChIP-seq_Cp190, wildtype, rep 2 - 33925006 unique reads - 144 bp, PE<br>GSM4405395 ChIP-seq_Cp190, CTCF[0] mutant, rep 1 - 24696710 unique reads - 144 bp, PE<br>GSM4405396 ChIP-seq_Cp190, CTCF[0] mutant, rep 2 - 31283806 unique reads - 144 bp, PE<br>GSM4405397 ChIP-seq_Cp190, Cp190[KO] mutant, rep 1 - 17591230 unique reads - 144 bp, PE<br>GSM4405398 ChIP-seq_Cp190, Cp190[KO] mutant, rep 2 - 21277088 unique reads - 144 bp, PE
Antibodies	Rabbit polyclonal antibodies against Drosophila CTCF and Cp190 proteins were generated for this study and are described in the Methods.
Peak calling parameters	The following was described in the Methods section:

	Paired end ChIP-seq reads were demultiplexed and mapped to the dm6 genome using a derivative of the fetchGWI tool. Only chromosomes 2, 3, 4 and X were used. ChIP-seq peaks were called using the R package csaw v1.16.1 using a window width of 20 bp and spacing of 10 bp (Lun & Smyth, 2015), ignoring duplicate reads. A background enrichment was evaluated as the median over all samples in the comparison of the average number of reads per 2 kb bins. Windows with less than 3-fold enrichment over background were filtered out. Data were normalized using the TMM method (Robinson & Oshlack, 2010) implemented in csaw. Differential binding analysis in csaw is based on the quasi-likelihood framework implemented in the edgeR package (Robinson et al., 2009). Results obtained on different windows were combined into regions by clustering adjacent windows. Combined p-values were evaluated for each region using csaw and Benjamini & Hochberg method was applied to control the false discovery rate. Regions with false discovery rate (FDR)<0.01 and |fold change|>2 were considered as differential binding regions. Genuine CTCF peaks were identified by differential analyses of ChIP-seq signals in WT versus CTCF[0] as being lower in the mutant samples relative to WT. Genuine Cp190 peaks were similarly identified by differential analyses of ChIP-seq signals in WT versus Cp190[KO] (WT Cp190 peaks) or in CTCF[0] versus Cp190[KO] (Cp190 peaks in CTCF[0]). Additional differential analyses were performed for Cp190 ChIP-seq signal in WT versus CTCF[0] (for Fig. 5a).
Data quality	(See details above.) CTCF peaks were obtained by performing differential binding analysis between the genotypes of interest and the control genotype CTCF[0]. Cp190 peaks were obtained by performing differential binding analysis between the genotypes of interest and the control genotype Cp190[KO].
Software	Micmap v2.20200223 (Iseli et al, 2007): https://github.com/sib-swiss/micmap<br>R v3.5.1 (R Core Team 2018): https://www.R-project.org/<br>csaw v1.16.1 (Lun and Smyth, 2014, 2015): https://bioconductor.org/packages/release/bioc/html/csaw.html<br>edgeR v3.22.5 (Robinson, McCarthy and Smyth, 2009): https://bioconductor.org/packages/release/bioc/html/edgeR.html

Flow Cytometry

Plots

Confirm that:

- [x] The axis labels state the marker and fluorochrome used (e.g. CD4-FITC).
- [x] The axis scales are clearly visible. Include numbers along axes only for bottom left plot of group (a 'group' is an analysis of identical markers).
- [x] All plots are contour plots with outliers or pseudocolor plots.
- [x] A numerical value for number of cells or percentage (with statistics) is provided.

Methodology

Sample preparation	Live Drosophila S2 cells were resuspended in phosphate buffered saline pH 7.5 48 hours after transfection.
Instrument	NovoCyte Flow Cytometer from Acea Biosciences
Software	R v3.5.1 (R Core Team 2018): https://www.R-project.org/
Cell population abundance	Approximately 10'000 cells were analyzed per sample, and transfection rates varied from 5-30% of all cells depending on the experiment. Average values from biological duplicate experiments were plotted in Figs. 4b-4c.
Gating strategy	The starting cell population (Drosophila S2 cells) was gated for FSC values between 6.2-6.8 (log10) and SSC values between 4.5-5.5 (log10). GFP and mCherry fluorescence was detected with the following excitation and emission gates: FITC 488 +530/30 and PE-TexasRed 561+615/20. A gate separating "transfected" from "untransfected" populations was manually set using untransfected control samples, and was kept constant for all samples. This gate is shown in Supplementary Figure 4a, and roughly corresponds to a cut-off of 4 (log10) for EGFP and for mCherry fluorescence.

- [x] Tick this box to confirm that a figure exemplifying the gating strategy is provided in the Supplementary Information.
